# How safe are our paediatric emergency departments? Protocol for a national prospective cohort study

**DOI:** 10.1136/bmjopen-2014-007064

**Published:** 2014-12-04

**Authors:** Amy C Plint, Amanda Newton, Antonia Stang, Maala Bhatt, Nick Barrowman, Lisa Calder

**Affiliations:** 1 Departments of Pediatrics, University of Ottawa, Ottawa, Ontario, Canada; 2 Children's Hospital of Eastern Ontario, Ottawa, Ontario, Canada; 3 Department of Pediatrics, University of Alberta, Edmonton, Alberta, Canada; 4 Department of Pediatrics and Community Health Sciences, University of Calgary, Calgary, Alberta, Canada; 5 Alberta Children's Hospital Research Institute, Calgary, Alberta, Canada; 6 Children's Hospital of Eastern Ontario Research Institute, University of Ottawa, Ottawa, Ontario, Canada; 7 Clinical Epidemiology Program, Ottawa Hospital Research Institute, Ottawa, Ontario, Canada; 8 Department of Emergency Medicine, University of Ottawa, Ottawa, Ontario, Canada; 9 Department of Emergency Medicine, The Ottawa Hospital, Ottawa, Ontario, Canada

**Keywords:** ACCIDENT & EMERGENCY MEDICINE

## Abstract

**Introduction:**

Adverse events (AEs), defined as unintended patient harm related to healthcare provided rather than an underlying medical condition, represent a significant threat to patient safety and public health. The emergency department (ED) is a high-risk patient safety setting for many reasons including presentation ‘outside of regular hours’, high patient volumes, and a chaotic work environment. Children have also been identified as particularly vulnerable to AEs. Despite the identification of the ED as a high-risk setting and the vulnerability of the paediatric population, little research has been conducted regarding paediatric patient safety in the ED. The study objective is to generate an estimate of the risk and type of AEs, as well as their preventability and severity, for children seen in Canadian paediatric EDs.

**Methods and analysis:**

This multicentre, prospective cohort study will enrol patients under 18 years of age from nine paediatric EDs across Canada. A stratified cluster random sampling scheme will be used to ensure patients recruited are representative of the overall ED population. A rigorous, standardised two-stage process will be used for AE identification. The primary outcome will be the proportion of children with AEs associated with ED care in the 3 weeks following the ED visit. Secondary outcomes will include the proportion of children with preventable AEs and the types and severity of AEs. We will aim to recruit 5632 patients over 1 year and this will allow us to detect a proportion of patients with an AE of 5% (to within an absolute margin of error of 0.6%).

**Ethics and dissemination:**

Ethics approval has been obtained from participating sites. Results will be disseminated through presentations, peer review publications, linkages with emergency research network and a webinars for key knowledge user groups.

**Trial registration number:**

This study is registered at Clinicaltrials.gov (NCT02162147; https://clinicaltrials.gov/ct2/show/NCT02162147).

**Strengths and limitations of this study**

- This will be the first prospective cohort study to generate an estimate of the risk and type of AEs, as well as their preventability and severity, for children seen in paediatric emergency department (EDs).
- This study is an essential first step to understand how to improve the safety of paediatric EDs and ultimately children's health outcomes.
- Many children are also seen in general or adult-oriented EDs and thus generalisability to these settings maybe limited.

## Introduction

Patient safety has been identified internationally as a healthcare priority.^1^ ^2^ Adverse events (AEs), broadly defined as unintended harm to the patient that is related to healthcare and/or services provided to the patient rather than the patient's underlying medical condition,^3^ represent a significant threat to patient safety and public health.^4–7^ Canadian data suggest 7.5% of hospitalised adults suffer AEs and one-third of these events are preventable.^6^ On a national level, this represents up to 23 750 preventable deaths per year among hospitalised adults.^6^ Recent data demonstrate that Canadian children are also at high risk with 9.2% of children admitted to hospital suffering an AE and almost half of these events are preventable.^7^ The economic burden of AEs is also high. From 2009 to 2010, the cost of AEs in the Canadian acute care system was estimated at $1.1 billion.^8^

To date, patient safety research has focused primarily on admitted patients. However, most Canadians, and especially children, are more likely to visit an emergency department (ED) than to be admitted to hospital. Of the over 16 million annual patient visits to EDs in Canada, only 9.2% result in admission.^9^ The ED is considered a high-risk setting for AEs due to variable provider experience, visits ‘outside of regular hours’, high patient volume, and a chaotic work environment characterised by frequent interruptions.^10–14^ The need for ED-based patient safety research is made more pressing by increasing demands on the ED system. ED crowding and long wait times have been linked to increased patient mortality,^15–19^ treatment delays^20–22^ and ambulance diversions.^23^

Only one systematic review of the prevalence, preventability, severity and types of AEs in the ED has been published.^24^ The proportion of ED patients who suffered at least one AE related to care provided in the ED varied widely between the studies included in this review, ranging from 0.16% to 6.9% of all patients. No study in the review examined how commonly AEs occurred among children presenting to the ED. The results of this review also suggest that a large proportion (36–71%) of AEs may be preventable and this is at least comparable to that reported in hospitalised patients (35–51%).^4–6^ ^25^ The review also suggested that the types of AEs that occur in the ED may be different than in hospitalised patients, and different between discharged and admitted ED patients.

Research has shown that children are particularly vulnerable to AEs.^7^ ^26^ ^27^ Reasons for this vulnerability include unique aspects of paediatric care such as weight-based medication dosing, children's inability to communicate complaints, and the physical and developmental characteristics of children that can affect treatment strategies, procedures and medication regimens.^27^ ^28^ For children treated in the ED, these vulnerabilities are in addition to the stressors inherent to the ED. Despite this, little research has been conducted on paediatric patient safety in the ED. We have no evidence even about how common AEs are among children seen and treated in EDs in children's hospitals. Such knowledge is an essential first step to understand how to improve the safety of paediatric EDs and ultimately children's health outcomes.

## Objectives

The objective of our multicentre, prospective cohort study is to generate an estimate of the risk and type of AEs, as well as their preventability and severity, for children seen in paediatric EDs across the Canada. AEs will be defined as unintended harm to the patient that is related to healthcare and/or services provided to the patient. Healthcare and/or services will include the actions of individual hospital staff (both acts of omission and acts of commission) as well as broader systems and care processes.^6^

## Methods

### Study population

#### Study design

This is a multicentre, prospective cohort study. We will enrol an estimated 5632 eligible patients over a 1-year period from nine paediatric EDs across Canada. Enrolled patients will be followed up to 3 weeks after their visit to identify AEs.

#### Study settings

This study will take place in the EDs of 9 of the 12 tertiary care children's hospitals in Canada: Janeway Children's Hospital (St. John's, Newfoundland and Labrador, Canada), Hôpital de St Justine (Montreal, Quebec, Canada), the Hospital for Sick Children (Toronto, Ontario, Canada), Children's Hospital of Eastern Ontario (Ottawa, Ontario, Canada), Children's Hospital London Health Sciences Centre (London, Ontario, Canada), Children's Hospital (Winnipeg, Manitoba, Canada), Alberta Children's Hospital (Calgary, Alberta, Canada), Stollery Children's Hospital (Edmonton, Alberta, Canada), and BC Children's Hospital (Vancouver, British Columbia, Canada). All are members of the a cross-Canada research network known as Pediatric Emergency Research Canada (PERC) and have individual annual ED censuses of between 35 000 and 72 000 for a total of approximately 470 000 patient visits/year.

#### Inclusion criteria

1. Age less than 18 years.
2. Patients from all paediatric Canadian Triage Acuity Scale categories (pedsCTAS (1) resuscitation (2) emergent (3) urgent (4) semiurgent, (5) non-urgent).

#### Exclusion criteria

1. Insurmountable language barrier that prevents informed consent and follow-up by telephone.
2. Children and families that will be unavailable for telephone follow-up in the 3 weeks after their ED visit (eg, no telephone in the home, travelling out of the country, etc).

### Outcome measures

#### Primary outcome measure

The primary outcome will be the proportion of patients who experience an AE related to ED care within 3 weeks of an ED visit. We will use the Canadian Patient Safety Institute definition of an AE as an event that results in unintended harm to the patient, and is related to the healthcare and/or services provided to the patient rather than to the patient's underlying medical condition.^3^ Healthcare and services will be defined to include the actions of individual hospital staff as well the broader systems and care process.^6^ ^7^ It will include harm related to acts of omission (failure to diagnose or treat) and commission (incorrect diagnosis or treatment, or poor performance).^6^ ED care will be defined as any care provided in the ED and will explicitly include care provided by ED specific staff (ie, staff physicians, nurses and allied healthcare providers) and care provided by consultants in the ED. Such a broad definition of ED care was chosen to reflect the overall patient experience and to be consistent with other studies.^12^ ^13^ The time frame for primary outcome estimation (3 weeks) is based on research documenting that the majority of AEs for adult-related ED care happen within 72 h of the ED visit, that 85% occur within 2 weeks, and that the remainder occur within 3 weeks.^12^ ^13^

#### Secondary outcome measures

1. *Proportion of patients experiencing a preventable AE:* A single physician reviewer will use a four-point Likert Scale (see online supplementary appendix 1) to determine preventability and must also identify the factor that made the event preventable.
2. *Clinical severity of AEs:* We will report only the most severe AE for a given child. We will take a broad, inclusive, patient-oriented perspective when considering what constitutes harm to the patient. As a result, we will classify severity according to two schemes. First, for all patients, we will utilise a previous published schema developed for studies including outpatients and report the clinical severity of AEs as: (1) an abnormality on laboratory testing, (2) ≤1 day of symptoms, (3) >1 day of symptoms, (4) non-permanent disability (5) permanent disability or (6) death.^12^ ^13^ ^29^ Non-permanent disability will be defined as temporary impairment of function lasting less than 3 months. Permanent disability will be defined as a permanent impairment of function. Given that we will have only 3 weeks of follow-up information for most patients, the degree of disability (non-permanent or permanent) will involve the physician reviewers’ clinical judgment. For admitted patients, we will also report clinical severity according to categories used by the Institute for Healthcare Improvement (IHI) trigger tool: (1) temporary harm to the patient requiring intervention, (2) temporary harm to the patient requiring initial or prolonged hospitalisation, (3) permanent patient harm, (4) intervention required to sustain life, or (5) death.^30^
3. *Types of AEs:* We will use a classification coding used in previous ED based studies:^12^ ^13^ (1) *Diagnostic issue:* documented signs, symptoms, laboratory tests or imaging not acted on, or an indicated diagnostic test not ordered; (2) *Management issue:* suboptimal management plans despite accurate diagnosis, or based on an inaccurate diagnosis; (3) *Unsafe disposition decision:* patient placed at unnecessary risk of experiencing death or major disability by being discharged from the ED or hospital; (4) *Suboptimal follow-up:* problems with follow-up arrangements led to the development of new symptoms, unnecessary prolongation of symptoms, an unscheduled return visit to the ED, or a subsequent unscheduled hospital admission (this could be due to inadequate availability of follow-up or due to inappropriate follow-up arrangements); (5) *Medication adverse effect:* patient experiences a symptom related to a medication regardless of whether the medication was appropriately prescribed or taken; (6) *Procedural complication:* patient experiences adverse consequences of a procedure; (7) *Nosocomial infection:* infection acquired in ED or in hospital.
4. *System response required for AEs:* The response will be classified as: (1) no treatment (symptoms only), (2) required medical/surgical intervention, (3) visit to medical doctor (MD) office, (4) visit to health laboratory/other health facility, (5) ED visit, (6) admission to hospital, (7) transfer to critical care, and (8) death. These previously published broad categories were chosen in order to address the effect of the AE at both the patient and healthcare system level.^12^ ^13^ ^29^ These categories are not exclusive.
5. *Proportion of patients for whom an AE is related to ED specific care* (vs consulting specialty service care provided in the ED or care provided after the child's ED visit).
6. *AEs related to care provided in the ED by consulting service*.
7. *AEs that occur within the 3-week time frame but are not related to care received in the ED* (including those related to in-hospital care and primary care).
8. *Patient and system level characteristics associated with AEs and preventable AEs*. These characteristics are further defined and described in online supplementary appendix 2.

### Sampling method

A stratified cluster-sampling scheme will be used to select shifts within each participating hospital. Patient presentations to the participating hospitals vary by time of day, time of the year and to a lesser extent day of the week. ED staffing, as well as hospital staffing levels and services, also vary by time of year, time of day and day of week. Studies have suggested that patients may have worse outcomes when presenting ‘outside of regular hours’.^31–33^ Owing to these factors, shifts within each hospital will be randomly sampled using a permuted block randomisation procedure to ensure balance by month, weekend/weekday and time (daytime 0800–1559, evening 1600–2359 h, and night 0000–0759 h).

### Overview of data collection procedures

Research assistants (RAs) collecting data in the ED, and research nurses completing telephone follow-up procedures, will use portable tablets (iPads) for real-time, web-based data collection.

#### Data collection at the index ED visit

RAs will be present in the ED for each randomised shift, and will aim to enrol consecutive patients presenting to the ED. An RA will approach families for consent using methods in keeping with site-specific ethics requirements. At all sites, for pedsCTAS level 2 (‘emergent’) patients at all sites, the RA will confirm with the clinical leader, responsible physician or bedside nurse that is appropriate for the RA to approach these families for consent/assent for the study. Patients requiring resuscitation room care (pedsCTAS level 1) will also be recruited for the study (as they may be at particular risk for AEs).^26^ For these patients, at all sites the RA will not approach the patient or their families until their medical condition is stable, the responsible ED staff has deemed it appropriate, and they have received verbal consent from families to approach. Once informed consent (from parents and capable adolescents) and assent (from the child where applicable) is obtained, the RA will collect demographic and personal healthcare data for the child, and healthcare systems level data related to their ED visit.

#### Specific data to be collected at the index ED visit

##### Patient level data

(1) age; (2) sex; (3) languages spoken by the child and family; (4) history of recent immigration (<5 years) to Canada; (5) presenting complaint documented on the ED record; (6) medical history (eg, chronic illnesses, hospitalisations, indwelling lines, etc.); (7) current medication use; (8) triage vital signs; (9) pedsCTAS score assigned to the visit; (10) discharge diagnosis.

##### Heath care system data

Given concerns that ED crowding has adverse outcomes for patients,^15–22^ we will attempt to measure crowding in several ways. For feasibility reasons, we will collect some data items at the midpoint of the hour of triage: specifically the number of patients in the ED, number waiting to be seen by a physician, number awaiting in-patient beds and average length of time between triage and registration. We will also collect data on the length of time for initial physician assessment of the patient, the area of the ED to which patient is triaged (participating EDs are divided into high and low acuity zones, most with separate space and staffing), ambulance off-load type (for patients who arrive by ambulance), type of first healthcare provider assessing the patient (eg, trainee, physician and nurse practitioner), training level of first physician assessing the patient (trainee vs staff), number of ED and consulting physicians involved in patient care, number of end of physician shift handovers for each patient, time between consult request and consultant arrival time to disposition decision, length of ED stay and discharge disposition. We will also collect information regarding the staffing of the ED during each shift (ie, number of nurses present during a study shift, number of staff physicians and number and level of medical trainees).

##### Other data tracked for each shift

We will track the number of patients presenting, and the number approached, eligible and consenting to the study. Given the chaotic nature of the ED, there will be patients during a study shift who will not be approached for the study by the RA—for these ‘missed’ patients we will gather age, sex, presenting complaint, triage level and discharge disposition (through retrospective chart review). Patients and/or parents who are approached for the study, but refuse consent, will be asked for consent to review of their medical record for the same data as ‘missed’ patients. These data will allow us to determine the generalisability of our study sample.

#### Data collection for patients discharged home following the index ED visit

A trained research nurse will contact all patients (and/or their parents) by telephone at 7, 14 and 21 days following their visit to administer a structured interview and identify patients with flagged outcomes.

#### Data collection for patients admitted to hospital at the index ED visit

A trained research nurse will contact all admitted patients (and/or their parents) by telephone (or in person if currently admitted) at 7, 14 and 21 days following their visit to administer a structured interview and identify patients with flagged outcomes. The research nurse will also screen the medical record of all admitted patients using the Canadian Pediatric Trigger Tool (CPTT) to assess for the presence of triggers during the first 3 weeks of hospital admission. If telephone follow-up reveals the patient had an ED visit or admission to another hospital, we will attempt to obtain these records for review (having obtained consent to do so at enrolment).

#### Data collection for enrolled patients ‘lost to follow-up’

For patients ‘lost to follow-up’, the research nurse will review the medical record for ED visits, outpatient visits, and admissions, and screen for triggers and flagged outcomes. The research nurse will also search, where permitted by provincial jurisdiction, the provincial coroner's database.

### Determining AEs

An established two-stage process, based on the seminal Harvard Medical Practice Study,^5^ will be used to identify AEs by first flagging outcomes and triggers to identify patients at high risk for AEs, and then reviewing their healthcare records.

#### Stage 1: identification of flagged outcomes and triggers

##### Flagged outcomes identified by telephone follow-up among discharged and admitted patients

A structured telephone interview modified from that used in other ED-based AE studies will be used to identify flagged outcomes on telephone follow-up.^12^ ^13^ A child will be considered to have a flagged outcome on telephone follow-up if they experience any of the following: new symptoms, worsening symptoms, new exacerbation of a chronic underlying illness, unscheduled visit to ED or health professional, unscheduled admission to hospital or death. We will also specifically elicit and consider as flagged outcomes any family or patient report of possible: medication problem, complication of care, miscommunication between staff, miscommunication between staff and family or patient, equipment problem or other issues that may have harmed patient.

##### Triggers identified on medical record review among admitted patients

In addition to telephone follow-up to determine the presence of flagged outcomes, children admitted to hospital will also have their medical record reviewed for the presence of any of 35 CPTT triggers^34^ within 3 weeks of the index ED visit. The CPTT is the first validated, comprehensive trigger tool available to detect AEs in acute care facilities for medical or surgical care, consists of 35 screening criteria to identify records with possible harm.^34^ The CPTT triggers include (but are not limited to) unexpected death, any code or arrest, unplanned admission, unplanned surgery or return to surgery, infection of any kind, transfer to higher level of care, wrong site/wrong procedure/wrong patient and dissatisfaction with care.

##### Triggers and flags for mental health patients

Mental health patients are typically excluded in AE studies, and specific methods for identifying AEs have not been published for this population. No trigger tool has been developed to identify AEs among admitted mental health patients, although the IHI has published a trigger tool for detecting adverse drug events (ADE) in the mental health setting.^35^ A Canadian review of patient safety in the mental health setting suggested eight domains that should be considered for mental health patients.^36^ We selected our flagged outcomes on telephone follow-up and triggers on medical record review based on these domains and the IHI mental health setting ADE trigger tool.^35^ We will include the following as additional flagged outcomes on telephone follow-up for patients whose index ED visit was for a mental health complaint: contact with mental health crisis lines, police, provincial child welfare agencies; attempted or actual self-harm; attempted or actual harm to others; attempted or actual harm by others. These flagged outcomes are in addition to those flagged outcomes outlined above on telephone follow-up. We will use the CPTT for admitted mental health patients, but will also consider the following to be triggers: any use of physical or chemical restraints, seclusion of the patient; any attempted harm to self, any attempted harm to or by others (including staff and other patients); any abscondment from the in-patient ward; and IHI mental health setting ADE triggers that are not also CPTT triggers.

##### Critically ill or deceased patients

It would be insensitive and unethical to approach families to participate in the study whose children die in the ED or who present with acute life-threatening injuries or illnesses that are not stabilised in the ED. Any children who die in the ED will be considered to have a flagged outcome and their medical record will be reviewed (stage 2 below) for an AE. Unstable children with life-threatening injuries or illnesses who were admitted to hospital will have their medical record screened by the research nurse using the CPTT. These children will be identified by the site research coordinator during his/her review of the ED registration list following each shift for ‘missed patients’ and entered into the study database.

##### Mental health patients

In order to not underestimate the risk of AEs among this vulnerable, high-risk population, we will take the following steps for children/youth with mental health complaints for whom it would not be appropriate to approach for consent (eg, critically ill children, such as children who are floridly psychotic or those who have presented with deliberate self-harm requiring resuscitation, and children who are behaviourally aggressive). For these children/youth, if they are admitted to hospital at their index ED visit we will complete at CPTT to look for ‘triggers’ in their record. For all children/youth in this group, we will also examine the electronic health record to determine whether the patient had a visit to the ED or admission in the 21 days following their index visit. If they are determined to have had a subsequent ED visit or admission, these patients will be considered to have flagged outcomes. Any patient with a flagged outcome or trigger will have their medical record will be reviewed (see stage 2 below) for an AE.

##### Preparation of case summaries

Any patient deemed to have a flagged outcome on telephone follow-up or a trigger on medical record review will have a case summary prepared by a research nurse. We will orient each site's research nurse on case summary creation. The case summaries are intended as narrative descriptions of what occurred at the index ED visit and the outcome. This technique is used in order to reduce the risk of handwriting recognition by examining the health record itself. These summaries will include: (1) patient demographics; (2) index ED visit details (such as date of visit, presenting complaint, vital signs, history of presenting illness, physical examination, investigations, treatment, etc.); (3) flagged outcome/trigger details (such as description of symptoms, date of return visits, ED visit details, and discharge or death summary details). Copies of the index ED visit, subsequent ED visits, discharge summary or death summary, as well as laboratory and other investigations (such as ECG and chest X-ray reports) will be accessible to the physician reviewers as required and will be de-identified.

#### Stage 2: identification of AEs

After standardised, comprehensive training, three ED physicians will independently review the case summary of each patient. They will then complete a computerised structured outcome assessment form. These reviewers will be blinded to patient name and sex, date and time of visit, treating physician, and study site. If the reviewers are unable to make their AE determinations based on the narrative case summary alone, they will have access to the key components of the medical record used in creating the case summary (blinded as outlined above). The reviewers will then be asked to independently determine whether the flagged outcome or trigger may be associated with healthcare management and rate their certainty of this determination on a six-point Likert scale used in previous studies (see online supplementary appendix 1).^4^ ^6^ ^7^ ^12^ ^13^ ^29^ On this six-point Likert scale, 1 represents a flagged outcome or trigger was most certainly due to disease and 6 represents that it was most certainly due to care. If two reviewers have a level of certainty ≥4, the outcome will be classified as an AE. If one reviewer reports a level of certainty ≥5, while the remaining report <4, the reviewers will discuss the case and reconsider their scoring. We have chosen to use three physician reviewers as previous research has shown that combining multiple reviews reduces uncertainty over the presence of an AE.^37^ To ensure that local practice patterns are considered during the review, the reviewer panel will include two site-specific reviewers and one reviewer from the coordinating site. One reviewer will determine whether the AEs were preventable using a four-point Likert scale with AEs classified as preventable if the reviewer has a certainty ≥3 (see online supplementary appendix 1) and can clearly identify the factor or factors that made the AE preventable. A single reviewer will also classify the AE type(s), severity and system response required. Previous work has shown that a single reviewer is adequate for this step.^37^

##### Identification of AE's related to care provided outside the ED

If the outcome determination as outlined above indicates the patient suffered an AE related to non-ED care, physicians from the appropriate service involved will also review these cases. For example, for all patients whose AE was deemed to be related to care provided during their inpatient stay, these cases will be further reviewed by a hospitalist paediatrician. These cases will be further discussed if consensus between the ED physician and service specific physician on outcome is not obtained.

##### Identification of AE's in patients with mental health entrance complaints

These cases will be reviewed by 2 ED physicians and a psychiatrist (rather than only ED physicians). The same methodology as outlined above will be used to determine if the patient suffered an AE.

### Data quality and training of research staff

Computerised, web-based data collection forms (through REDCap) will be used throughout this study to ensure complete data entry. Portable tablets (iPads) will be used to collect all data and embedded logic safeguards will ensure variables are entered within predetermined limits. Warning messages will prompt the user for any incomplete fields. Standardisation of study methods will be achieved through training in all activities and outcome tools used by research staff. Research nurses applying the CPPT for admitted patients will be trained using a standard set of medical records and a training manual. Interobserver reliability of use of the CPTT will be assessed during the training session and on a random selection of 10% of records throughout the study. Case summaries prepared by the site research nurse will be reviewed until 10 consecutive summaries accurately reflect the medical record and then 5% will be randomly reviewed for integrity. If case summaries are found to have discordant information, remedial instruction will be given and all case summaries will be reviewed until 10 consecutive records are accurate, at which time the random screening will recommence. Physician outcome reviewers will receive training in definitions, use of cases for practices and discussion of discrepancies in outcome coding. Interobserver reliability of physician determination of AE will be assessed during the training session and on a random selection of 5% of records throughout the study.

### Sample size

The primary outcome is a proportion, the occurrence of AEs related to ED care among a cohort of ED patients. In our recent systematic review, we found that 0.16–6.0% of ED patients had an AE related to ED care.^24^ Studies using medical record review determination of AEs, versus surveillance or active reporting, consistently find the highest proportion of patients with AEs. Among the highest quality ED-based studies, the AE rate ranged from 5% to 6.0% (primarily adult patients). The only Canadian study of AE among admitted children found that overall 9.2% of children had an AE. While it would be ideal to consider clustering by shift in our sample size calculation, it is not possible due to lack of data. Because of this and also given the range of AEs reported among ED patients, we have chosen to be conservative in our sample size estimates. We will aim to enrol 5632 patients over a 1-year period and assume a 10% loss to follow-up. This will allow us detect a proportion of patients with an AE related to care provided in the ED of 5% to within an absolute margin of error of 0.6% (with 95% CI).

### Data analysis

#### Outcome analysis

Descriptive statistics will be used to describe enrolled patients. The primary outcome, the proportion of children with AEs related to ED care, will be reported with 95% CIs, accounting for the stratified cluster sampling design using SAS PROC SURVEYFREQ. Estimates of design effect will be examined. Secondary outcomes will be similarly estimated. To explore the association with AE and preventable AE of patient-level and system-level characteristics (together and separately) we will use univariate methods (PROC SURVEYFREQ) and multiple logistic regression (PROC SURVEYLOGISTIC). With sample size at follow-up of 5069, and an anticipated 5% rate of AEs, we expect approximately 253 AEs. Following the recommendation that approximately 10 events are required for each variable included in a multivariate model^38^ will allow us to include up to 25 predictor variables. Similar analyses will also be performed for preventable AEs. If 50% of AEs are preventable, we would expect approximately 126 preventable AEs, allowing us to include 12–13 predictor variables. Factors significant at the two-sided p<0.10 level on univariate analysis will be considered for inclusion in the multivariate model. When variables are highly correlated, the less clinically relevant ones will be omitted. Patient characteristics (see online supplementary appendix 2) to be examined include age, sex, language, immigration, triage level, time, weekday/weekend presentation, discharge disposition, pre-existing mental health condition, pre-existing health condition, use of any prescription medications and complex illness.^7^ System-level factors (see online supplementary appendix 2) measurable for each individual patient include length of time to see physician, number of ED staff physicians involved in that patient's care, number of ED physicians (trainees and staff) involved in that patient's care, location within the ED, need for a consultation, number of services consulted, level of physician initially managing the patient and delay to ambulance off-load for patients arriving by ambulance. System level factors pertaining to the environment of the ED around the time of patient arrival that may be markers of crowding include the overall ED census, number of patients waiting to be seen, number of patients awaiting in-patient beds and average time between patient triage and registration. Since these environmental factors may depend on the size of the ED, we will also consider as a variable the number of beds in the ED.

#### Subgroup analyses

Estimation of proportions of patients with AEs and secondary outcomes will be repeated for the following groups: (1) medical/surgical patients, (2) mental health patients, (3) admitted versus discharged patients, (4) children <1 year of age, (5) patients with complex medical conditions and (6) high acuity patients (pedsCTAS 1 and 2).

## Ethics and dissemination

### Ethical considerations

There is minimal risk to patients for this study as it is observational and will not interfere with patient care. Risks exist in the realm of privacy of data. Research personnel will take all necessary steps to ensure that data remains secure and privacy is maintained. No patients will be enrolled in the study without written informed consent from the patient or parent/substitute decision-maker. Assent will also be obtained from participants as appropriate by age. There will be no incentives offered to potential participants or their parents/substitute decision-maker to take part in the study. Research ethics board approval has been obtained from all sites participating in this study. Experienced nurses will complete telephone follow-up and if concerned about a child's medical status, will instruct the parent/child to obtain appropriate follow-up care. Serious AEs will be brought to the attention of the appropriate administrators. Study investigators will not disclose AEs directly to the study participants or their families but will provide sufficient information to appropriate administrators such that each study site's hospital specific policies and any regulatory body policies regarding AEs may be followed.

### Knowledge translation

A multifaceted knowledge translation strategy will be used. Through PERC and PERN (Pediatric Emergency Research Networks)^39^ we will disseminate the results of this study to a broad range of stakeholders. These networks represent not only paediatric ED researchers worldwide (with 122 hospitals represented within the 5 networks of PERN), but also include practicing ED clinicians, and local, provincial, and national healthcare administrators as members. PERC is closely tied to Networks of Centers of Excellence funded TREKK (Translating Emergency Research Knowledge for Kids).^40^ TREKK is a unique partnership and knowledge exchange between 36 general EDs across Canada and 12 PERC sites. The annual PERC, TREKK and PERN meetings and internal communication structures of these organisations will allow knowledge gained in this study to be widely and rapidly disseminated.

To further ensure distribution of study results, the principal investigator, in conjunction with the Canadian Association of Paediatric Health Centres (CAPHC), will lead a webinar series aimed at key knowledge user groups: healthcare providers and funders, medical directors and administrators of acute care facilities, organisations representing knowledge users in paediatric and emergency medicine, patient safety organisations and networks, and paediatric, emergency, and safety researchers. Webinars will: (1) raise awareness of study results and opportunities for future involvement, (2) provide an opportunity to discuss implications for local clinical practice settings, and (3) seek input on how to further translate study findings into clinical, policy and research recommendations. Emphasis will be placed on translating findings into clinical, policy and/or research recommendations. We will use webinar feedback to craft a set of initial recommendations, which will be refined by a Steering Committee. This Committee will consist of key members of the organisations outlined above, knowledge users who self-identify during the webinars and parents. Parent representation will be sought through the Canadian Family Advisory Network. An in-person Steering Committee meeting to establish a final set of recommendations will situate our study's findings as relevant and tangible outcomes, and provide a planning base for nation-wide initiatives that build on current evidence and clinical practices. Study findings will be presented at international paediatric, emergency medicine, and patient safety conferences and seek publication of study results in peer-reviewed journals. Finally, all study participants and their parents/substitute decision-makers will also be sent a lay summary of the final study results.

## Supplementary Material

Author's manuscript

Reviewer comments
