# Supplementary material for: How safe are our paediatric emergency departments? Protocol for a national prospective cohort study
Source: BMJ Open. 2014 Dec 4;4(12):e007064. doi: 10.1136/bmjopen-2014-007064 (PMC4256537; doi:10.1136/bmjopen-2014-007064)
Supplement: Reviewer comments [file bmjopen-2014-007064.reviewer_comments.pdf]

PEER REVIEW HISTORY

BMJ Open publishes all reviews undertaken for accepted manuscripts. Reviewers are asked to complete a checklist review form (**http://bmjopen.bmj.com/site/about/resources/checklist.pdf**) and are provided with free text boxes to elaborate on their assessment. These free text comments are reproduced below.

ARTICLE DETAILS

**TITLE (PROVISIONAL)**	How safe are our pediatric emergency departments? Protocol for a national prospective cohort study
**AUTHORS**	Plint, AMy; Newton, Amanda; Stang, Antonia; Bhatt, Maala; Barrowman, Nick; Calder, Lisa

This article was not externally reviewed at BMJ Open. Protocols that have been independently assessed prior to submission to BMJ Open are usually fast-tracked to publication on the grounds that further substantial changes will not be possible. This independent assessment will usually be external, independent review for both a substantial grant award from a non-commercial or government funder and ethics approval.
